# Precues’ elevation of sensitivity is not only preattentive, but largely monocular

**DOI:** 10.3758/s13414-018-1564-1

**Published:** 2018-07-09

**Authors:** J. A. Solomon, M. J. Morgan

**Affiliations:** Centre for Applied Vision Research, City, University of London, London, UK

**Keywords:** Space-based attention, Precueing

## Abstract

Visual sensitivity can be heightened in the vicinity of an appropriate precue. Experiments with multiple, noninformative precues suggest that this facilitation should not be attributed to focal attention. The number of simultaneously appearing precues seems to be irrelevant; contrast thresholds are lowest for targets that appear in a precued position. Here we report that precues become less effective when they and the target are delivered to different eyes. We conclude that the mechanism responsible for such heightened sensitivity has largely monocular input.

**Electronic supplementary material:**

The online version of this article (10.3758/s13414-018-1564-1) contains supplementary material, which is available to authorized users.

Posner, Nissen, and Ogden (1978) showed that, in certain circumstances, reaction times (RTs) for detecting a target were lowered when the target was preceded by a visual cue (a “precue”) to its position. Specifically, those circumstances depended on the type of cue. Endogenous cues are those appearing near fixation. These cues shorten reaction times when the target appears roughly one second after the cue. Exogenous cues, on the other hand, are those appearing near the target. Exogenous cues shorten reaction times when the target appears roughly 100 ms after the cue onset.

Posner’s findings have been replicated using other types of dependent variable. For example, Bashinski and Bacharach (1980) confirmed a precueing advantage for visual sensitivity. Criterion orientation–identification could be attained with a lower stimulus contrast when the visual target appeared on the precued side of fixation. This was merely the first published experiment to assess precueing’s effect on visual sensitivity. (See Table 1 in Solomon, 2004, for a list of 23 such experiments.)

Jonides (1981) pioneered the use of noninformative precues. In Jonides’ paradigm, a single pre-cue appeared randomly near one of eight possible target positions, and the target appeared in that position on exactly one-eighth of the trials. The cue therefore offered no information to an ideal observer. Nonetheless, reaction times to precued targets were shorter than those to uncued targets.

Wright (1994) extended Jonides’s noninformative cueing paradigm, precueing two, four, and all eight of the possible target positions simultaneously. His observers were required to report whether a tilted line segment was tilted clockwise or counterclockwise of vertical, as quickly and as accurately as possible. In this experiment Wright investigated two independent variables: the number of exogenous precues and their positions. The dependent variable was reaction time. Wright’s findings strongly suggested no effect of either independent variable. All that mattered was whether or not the target’s position was cued. If so, reaction times were relatively short. If not, they were relatively long. Wright concluded that exogenous precues can indeed facilitate visual performance, but they need not necessarily engage focal attention to do so.

In the same way that Bashinski and Bacharach (1980) confirmed Posner et al.’s (1978) original findings using contrast threshold as a dependent variable, Solomon (2004) confirmed Wright’s (1994). There seemed to be no capacity limit (unless it was greater than eight cued positions) for the facilitatory effect of precues on visual sensitivity. This lack of a capacity limit strongly suggests that the mechanism responsible for lowering contrast thresholds must occupy a relatively low level or early position in the visual processing hierarchy, as it is not subject to the bottleneck of selective attention.

To better localize the mechanism responsible for exogenous cueing, Self and Roelfsema (2010) compared the effect of precues presented to the same eye as a monocularly presented target to the effect of precues presented to the other eye. Both types of precue shortened reaction times, but those delivered to the same eye as the target were most effective when the cue–target onset asynchrony (CTOA) was 0.050 s, whereas cues delivered to the other eye were most effective with a CTOA of 0.400 s, leading Self and Roelfsema to infer that two different mechanisms could contribute to exogenous cueing, one of which had input from monocular levels of visual processing.

In this article, we combine Self and Roelfsema’s (2010) idea of comparing “same-eye” and “different-eye” precues with the paradigm used by Solomon (2004), to see whether or not the unlimited capacity he reported should be attributed to the early (monocularly driven) mechanism. Prior to a discussion of our new experiments, it should be noted that Self and Roelfsema argued against unlimited capacity at the monocular level. However, that argument was based on a comparison between reaction times for monocularly presented targets appearing after a precue had been delivered to the same eye and after otherwise identical precues had been delivered to both eyes (the latter were somewhat longer). They did not try comparing single, monocular cues with multiple monocular cues. Consequently, that became the focus of our Experiment 1, wherein we compared the effects of precues that were delivered to the same eye as the target with precues that were delivered to the other eye. To anticipate, Solomon’s (2004) unlimited-capacity results were replicated only when the cue and target were delivered to the same eye. There was some interocular transfer of the cueing effect, but not much.

## Experiment 1

### Method

All four observers were highly experienced with psychophysical methods, but only the two authors were aware of this study’s purpose. This study used (some) different hardware and software (available at www.staff.city.ac.uk/~solomon/CueingTransfer.zip) from that used in Solomon (2004), but we attempted to follow that earlier study’s methods as closely as possible.

The stimuli were displayed on a Sony GDM-F520 monitor. A video signal with 14-bit precision was attained using the Bits# hardware (Cambridge Research Systems, CRS). Ferro-optical goggles (also from CRS) were used to separate the left eye’s and right eye’s images. The frame rate of the monitor was 120 Hz (i.e., 60 Hz for each eye). All stimuli were displayed on a mean-gray background that was exactly halfway between the monitor’s minimum and maximum luminances. Initially, those values were 42.57 and 153.8 cd/m^2^. However, following an involuntary “security” update to the graphics drivers, minimum and maximum luminances rose to 51.58 and 172.5 cd/m^2^. The data from the two authors were collected with the initial values given here; the data from the other two observers were collected with the latter values.

**Fig. 1:**
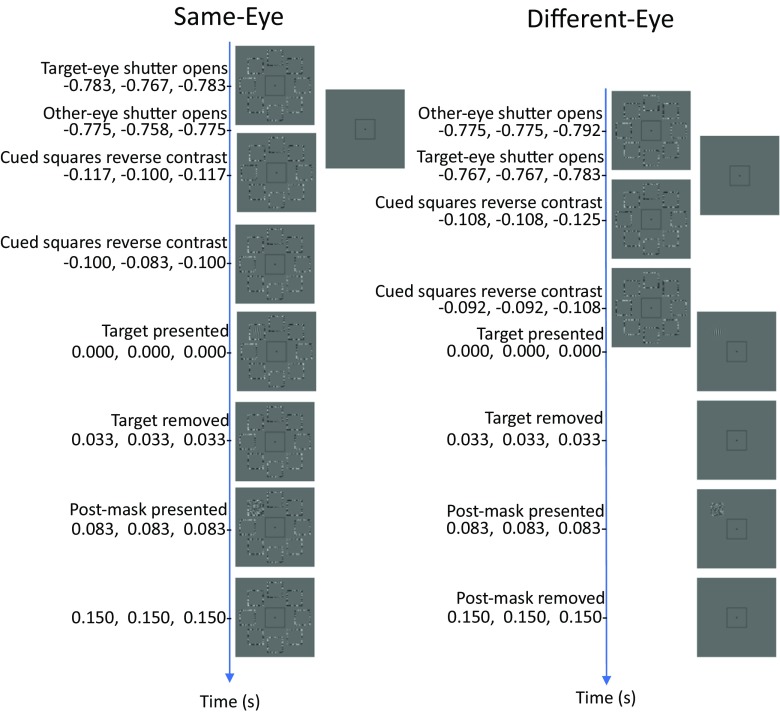
Example trial sequences. Timings relative to target onset appear as triplets: {Exp. 1, Exp. 2, Exp. 3}. The intervals between these timings are not represented to scale. The central black square and fixation spot were delivered to both eyes throughout the experiment. The random noise surrounding all eight positions was delivered to one eye. In half the trials, the target Gabor pattern and its random-noise postmask were delivered to the same eye. In the other trials, the target and its postmask were delivered to the other eye. The random noise surrounding zero, one, two, four, or all eight positions reversed in contrast twice, providing no information regarding the position of the target. (In these examples, only the top-left position is precued.) A postmask appeared at the target position 0.083 s after target onset (all conditions, all experiments). Observers were instructed to report the orientation of the target’s tilt (clockwise or counterclockwise with respect to vertical).

Figure 1 illustrates the central 480 × 480 pixels of the 640 × 480 display. At the 1.09-m viewing distance, there were 32 pixels per degree of visual angle. Eight positions, each 5.25° from fixation, were circumscribed with squares of maximum-contrast binary noise (check size: 4 × 4 pixels). Each side of each noise square was 94 pixels long and 4 pixels wide (see Fig. 1). These noise squares were presented to one eye only. On each trial the observer saw the sequence: cue, target, postmask. The cue was a contrast reversal occurring 0.667 s after the preceding response (a button press, indicating the target orientation); the noise around one, two, four, all, or none of the positions reversed contrast, and 0.017 s later, it reversed back. A Gabor target then appeared.

The contrast reversals that defined our precues were easily detected. However, it is probable that some observers would been unable to report which locations were and were not cued with perfect accuracy. Since we had no hypotheses about awareness of (or memory for) the precues themselves, we did not collect such measures.

Note that our apparatus requires an even number of frames between the onset of the precue and that of any target delivered to the same eye, and it requires an odd number of frames between the onset of the precue and that of any target delivered to the other eye. For the latter condition, we used the same number—13—used by Solomon in his earlier (2004) experiment (i.e., 0.108 s; see Fig. 1). For the former condition, we used 14 (i.e., 0.117 s; see Fig. 1).

A postmask of maximum-contrast binary noise (check size: 1 pixel) appeared 0.083 s after the target, filling the square at the target position only. Finally, 0.150 s after the target first appeared, the postmask disappeared, leaving only the fixation spot, noise frames, and a black square (designed to aid proper binocular convergence) visible onscreen.

Observers completed seven blocks of trials on each day of testing. In total, these seven blocks took roughly 1 h to complete. The order of blocks was randomized. Five of the blocks contained five trials in each of 32 conditions, for a total of 160 trials per block. In all conditions, the central black square and fixation spot were delivered continuously to both eyes. In Conditions 1–16, the random noise surrounding all eight positions, as well as the target Gabor pattern and its random-noise postmask, were delivered to the same eye. In Conditions 17–32, the random noise was delivered to one eye, and the target and its postmask were delivered to the other. Further details are given in Table 1. In all conditions, the cued positions were randomly determined on each trial. This protocol ensured no correlation between the position(s) of the cues and the position of the target.

**Table 1:** Conditions investigated in experiments

Condition Index in Experiment 1	Condition Index in Experiments 2 & 3	Number and Details of Noninformative Precues
1	1	single (same-eye) cue (target position)
1 + *i*, 1 ≤ *i* ≤ 7	1 + *i*, 1 ≤ *i* ≤ 7	target *i* positions CCW of single (same-eye) cue
9		two (same-eye) cues (one in target position)
10–12		two (same-eye) cues (none in target position)
13		four (same-eye) cues (one in target position)
14		four (same-eye) cues (none in target position)
15		eight (same-eye) cues
16		no (same-eye) cues
17	9	single (different-eye) cue (target position)
17 + *i*, 1 ≤ *i* ≤ 7	9 + *i*, 1 ≤ *i* ≤ 7	target *i* positions CCW of single (different-eye) cue
25		two (different-eye) cues (one in target position)
26–28		two (different-eye) cues (none in target position)
29		four (different-eye) cues (one in target position)
30		four (different-eye) cues (none in target position)
31		eight (different-eye) cues
32		no (different-eye) cues

CCW, counterclockwise.

The target contrast in each condition was controlled by the QUEST algorithm (Watson & Pelli, 1983), which converged to threshold, as defined below. QUEST staircases were initialized at the beginning of the first block of 160 trials, but they were resumed in the subsequent blocks, providing 25 trials per staircase per condition per day.

One of the two remaining blocks of trials consisted of 25 trials of Condition 1, not interleaved with anything else, to determine performance when a totally valid cue was delivered to the same eye as the target. The other remaining block consisted of 25 trials of Condition 17, to determine performance when a totally valid cue was delivered to the other eye. All observers understood which blocks contained noninformative cues and which blocks contained totally valid cues. On each trial in all blocks, the eye to which the target was delivered was determined at random and independently.

Observer J.A.S. used the same target (a Gabor pattern with wavelength *λ* = 0.19° and space constant *σ* = 0.45°) that he had used in the previous study (Solomon, 2004). As in that study, preliminary measurements suggested large individual differences in contrast sensitivity. To accommodate these differences, the target’s wavelength was increased to *λ* = 0.38° for the other three observers. J.A.S. completed all seven, and M.J.M. completed five days’ testing prior to the aforementioned update to the graphics driver. Observers M.L. and P.C. each completed seven days’ testing after the update.

### Results

For each condition, contrast thresholds for orientation identification were estimated by maximum likelihood (Watson, 1979), fitting a Weibull function to all of the psychometric data from each observer:

1 $$ \Psi \left(t;{t}_0,\beta \right)=0.5+0.49\left(1-\exp \left[-{\left(t/{t}_0\right)}^{\beta}\right]\right) $$

In this expression, the accurate proportion of responses Ψ, is a function of the target contrast *t* and the parameters *t*_0_ (threshold) and *β* (psychometric slope). Although both of the latter parameters were allowed to vary freely in the fitting procedure, the QUEST algorithm was designed to constrain estimates of threshold only. Since there was no indication that threshold varied systematically with cue–target separation in Conditions 2–8, the psychometric data from these conditions were pooled, and the threshold was estimated again. Similarly, the psychometric data from Conditions 18–24 were pooled (but see Fig. 4 below), as were the data from Conditions 9–11 and those from Conditions 25–27. Parametric bootstrapping (Efron & Tibshirani, 1993) was employed to determine the standard errors of these maximum-likelihood estimates of threshold.

**Fig. 2:**
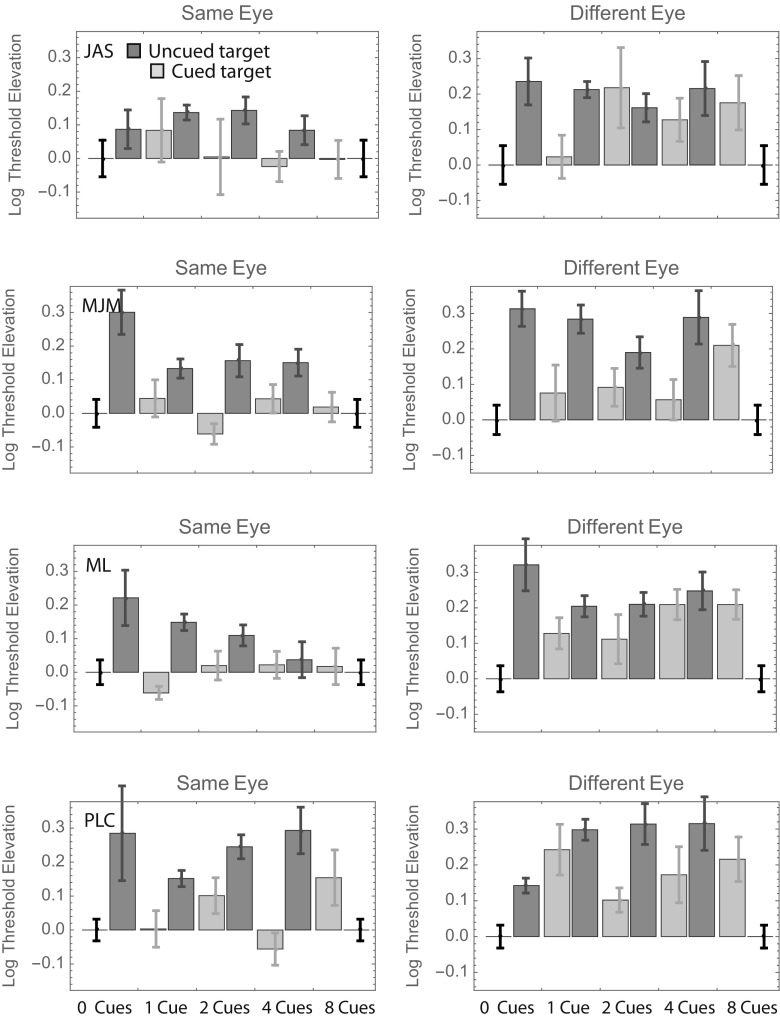
Log threshold contrast elevations with respect to the 100%-valid same-eye condition in Experiment 1. The error bars framing each panel are associated with the 100%-valid same-eye condition itself, and different color bars show the results from conditions in which the target appeared at precued and uncued positions. Error bars show ± 1 *SD* from the parametric bootstrap distribution. Not shown are log threshold elevations from the 100%-valid different-eye condition; for J.A.S., M.J.M., M.L., and P.C., these values were – 0.05, – 0.10, + 0.04, and + 0.17, respectively. NB: Negative “elevations” indicate lower thresholds.

Log threshold elevations for each observer are shown, with their corresponding standard errors, in Fig. 2, where a value of 0 indicates the threshold in the 100%-valid same-eye condition. (Standard errors for this threshold are shown at either end of the horizontal axis—i.e., where the threshold elevation is zero.) Different color bars show the threshold elevations obtained when the target did and did not appear at a cued position.

**Fig. 3:**
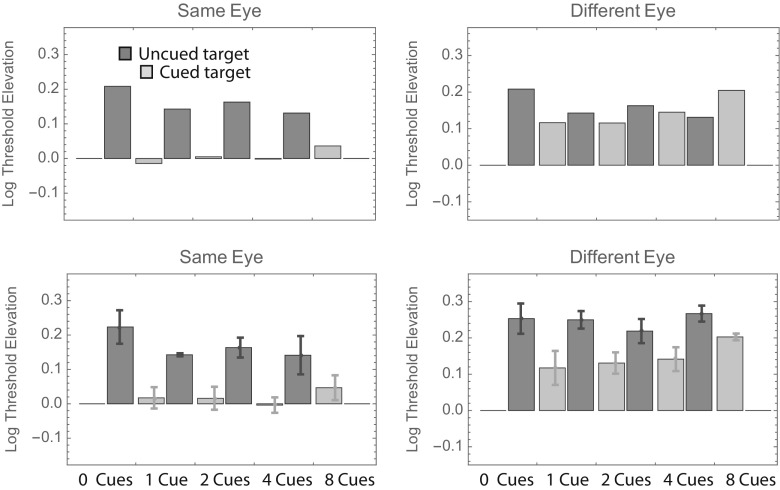
Results of Experiment 1, summarized in two ways. In the top two panels, each bar shows the weighted average log threshold elevation across observers. The weights are proportional to the inverse of the standard error. In the bottom two panels, each bar shows the unweighted average log threshold elevation across observers. Error bars show ± 1 *SE* (i.e., ± 1 *SD*/$\sqrt{N}$, where *N* = 4)

Visual inspection of Fig. 2 reveals that the uncued bars tend to be taller than the cued bars (i.e., the orientation of cued targets can be identified with less contrast), and the right-hand (“different-eye”) bars tend to be taller than the left-hand (“same-eye”) bars. Beyond those gross generalizations, it is hard to discern any overall pattern that is common to all observers. To facilitate such an analysis, we pooled the data across observers in two ways. The top panels in Fig. 3 show weighted average log threshold elevation across observers, where the weights are proportional to the inverse of the standard error. The bottom panels show the unweighted averages ± 1 standard error. Both summaries suggest a threshold elevation of ~ 0.15 log units when uncued targets appear in the same eye as the cues. The number of cues seems to have little effect.

The aforementioned results for the same-eye cues are consistent with those reported by Solomon (2004). The unweighted averages (lower right panel) suggest increased elevations in all conditions in which targets appear in the other eye. Indeed, a fully factorial, three-way analysis of variance (ANOVA; Same or Different Eye × Number of Precues × Cued or Uncued Target) with each observer’s threshold elevation (with respect to the 100%-valid same-eye condition) treated as a repeated measurement suggests significant main effects of same versus different eye (*p* < 10^–6^) and cued versus uncued target (*p* < 10^–8^), but no significant effect of number of precues (*p* > .2) nor any significant interactions (all *p*s > .15).

It is noteworthy that the average “0-cue” same-eye threshold is almost identical to the “0-cue” different-eye threshold. On average, these conditions produced the highest thresholds in Experiment 1. We also conducted another fully factorial three-way ANOVA on these data, this time using each observer’s threshold elevation with respect to the average of his two 0-cue conditions as a repeated measurement. The results were similar to those from the previously mentioned ANOVA: significant main effects of same versus different eye (*p* < .001) and of cued versus uncued target (*p* < .001), but no significant effect of number of precues (*p* > .7) nor any significant interactions (all *p*s > .6). Together with the main effect of cue, the lack of interactions leads us to conclude that all targets (even the uncued ones) received some facilitation from our precues. This general facilitation could be described as a spatially unspecific temporal warning effect. Note, however, that this effect was much smaller when the cues and target were delivered to different eyes. The average threshold depression (i.e., the opposite of threshold elevation) was 0.151 log units with intra-ocular cues, and 0.036 log units with interocular cues. Thus, we concluded that precues become 76% less effective when they and the target are delivered to different eyes.

Note that the two 0-cue conditions were not the same. In all same-eye conditions, the noise frames around each position were delivered to the same eye as the target. In all different-eye conditions, they were delivered to the other eye. Consequently, there was some risk that the different-eye noise frames could have exerted stronger lateral masking than the same-eye noise frames (e.g., Meese & Hess, 2005). Fortunately, that does not seem to have been the case using the present stimulus geometry. There must be some other reason why the thresholds were higher for cued targets in the different-eye conditions than they were for cued targets in the same-eye conditions. A monocular component of the precueing mechanisms is one interpretation, but there are others.

**Fig. 4:**
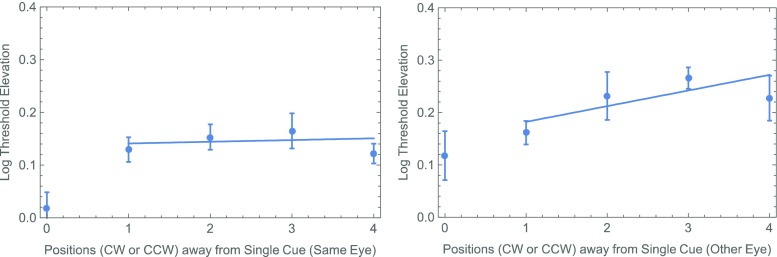
Summary of thresholds with a single noninformative cue from Experiment 1, showing a systematic effect of proximity when a single position is cued and the target is delivered to the other eye (right panel), but not when cue and target are delivered to the same eye (left panel). Each of these points represents the unweighted average (± 1 standard error) across observers. All threshold elevations (i.e., in both panels) were calculated with respect to the 100%-valid same-eye condition. The straight lines are (least-squares) best fits through the data from uncued positions.

One possibility is the slight (1 frame = 8 ms) difference in the CTOAs. Experiment 2 was designed to test this possibility. At the same time, we wanted to replicate the surprising result of our analysis of cue–target proximity in the different-eye conditions. Specifically, as is shown in Fig. 4 (right panel), there seems to be a systematic rise in threshold with the distance between a single cue and a different-eye target. Even when the thresholds for cued targets (zero separation) are excluded, an effect is apparent of the cue–target distance on the threshold, which does not quite reach statistical significance: *F*(1, 26) = 3.30, *p* = .081. As in our previous experiment with natural (i.e., binocular) viewing, no such effect was found with same-eye targets (Fig. 4, left panel): *F*(1, 26) = 0.06, *p* = .809.

Before moving on to Experiment 2, we address the matter of the psychometric slope, which has the potential to discriminate between popular models of detection, not only in the absence of noise, but also in the presence of noise masks that are equivalent to (but possibly more intense than) the detection-limiting noise internal to the visual system (e.g., Solomon & Tyler, 2017). First and foremost, without significant modification, none of those models can be applied to the task we study here. Our target orientations (± 11.5° with respect to vertical) were too similar to support the models’ shared assumption that different targets would stimulate independent mechanisms. Indeed, previous research (Thomas & Gille, 1979) suggests that a 20° difference in orientation might not be sufficient for identification thresholds to be as low as the detection thresholds, even if the targets were to appear at fixation. Furthermore, our poststimulus mask could not be considered equivalent to any noise within the visual system, since it was restricted to very specific spatial and temporal coordinates. (It consequently eliminated all extrinsic uncertainty regarding where and when the target would appear; intrinsic uncertainty, of course, is another matter.)

Nonetheless, we computed maximum-likelihood estimates of the psychometric slopes in all conditions.^1^ The foregoing reservations notwithstanding, we might expect observers to harbor greater uncertainty regarding uncued targets, which could manifest as steeper psychometric functions. The results, however, are not consistent with this hypothesis. Indeed, and prima facie at odds with Dosher and Lu’s (2000) report that “threshold ratios” (indices of psychometric slope) remain unaffected by the validity of an exogenous cue, our analyses indicate that the psychometric function is steeper for cued than for uncued targets when data are pooled across cue–target distances.

Recall, however, that any effect of cue–target distance on uncued thresholds (e.g., Fig. 4, right panel) must necessarily result in a reduction of the psychometric slope (e.g., Solomon & Morgan, 2006). When our data were segregated on the basis of cue–target distance, the cued slopes remained steeper in some experimental conditions (Exp. 3: different-eye; Exp. 2: both conditions), but not others (Exp. 3: same-eye; Exp. 1: both conditions).

## Experiment 2

### Method

All four observers from Experiment 1 participated in Experiment 2. Again, only the two authors were aware of this study’s purpose. In this experiment we used the same hardware (with minimum and maximum luminances of 51.58 and 172.5 cd/m^2^) as in Experiment 1.

As is indicated in Fig. 1, the different-eye timings were identical to those in Experiment 1 (with CTOA = 0.108 s), but the CTOA decreased by two frames, from 0.117 to 0.100 s, for the same-eye conditions. Exactly one position was precued in each trial.

The observers completed six blocks of trials on each day of testing. In total, these six blocks took only a half hour to complete. The order of blocks was randomized. Five of those blocks contained five trials in each of 16 conditions, for a total of 80 trials per block. In Conditions 1–8, the random noise surrounding all eight positions, as well as the target Gabor pattern and its random-noise postmask, were delivered to the same eye. In Conditions 9–16, the target and its postmask were delivered to the other eye. Further details are given in Table 1. As in Experiment 1, this protocol ensured no correlation between the position(s) of the cues and the position of the target.

The remaining block of trials consisted of 25 trials of Condition 1, not interleaved with anything else, again to determine performance when a totally valid cue was delivered to the same eye as the target.

In Experiment 2, all observers used the same target: a Gabor pattern with wavelength *λ* = 0.38° and space constant *σ* = 0.45. All observers completed seven days’ testing.

### Results

**Fig. 5:**
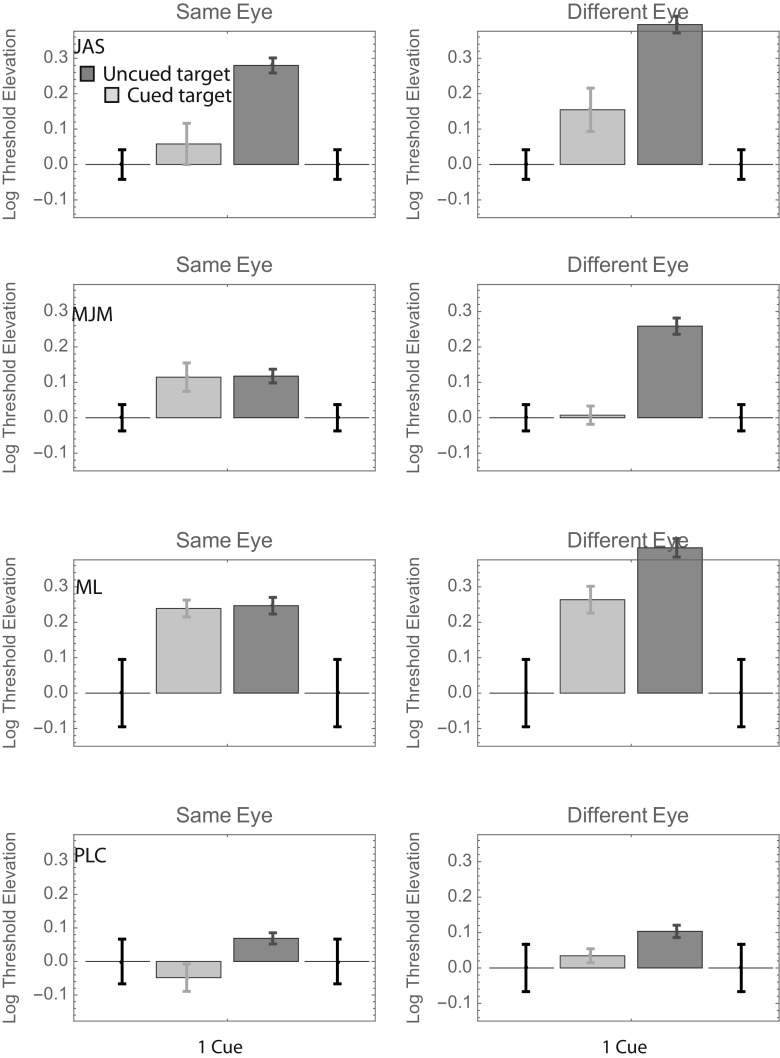
Log threshold contrast elevations with respect to the 100%-valid same-eye condition in Experiment 2. The format is analogous to that in Fig. 2: Different color bars show the results from conditions in which the target appeared at precued and uncued positions.

As for Fig. 2, we pooled the data from Conditions 2–8 when computing the uncued bar for each left panel of Fig. 5. Similarly, the data from Conditions 10–16 were pooled when computing the uncued bar for each right panel. As in Experiment 1, here it would be fair to say that most of the uncued bars are taller than their corresponding cued bars (although this difference was nonsignificant in two out of the eight cases), and most different-eye bars are taller than most same-eye bars (in one case—M.J.M. uncued—the difference is significant in the other direction; i.e., the threshold was greater for same-eye targets).

**Fig. 6:**
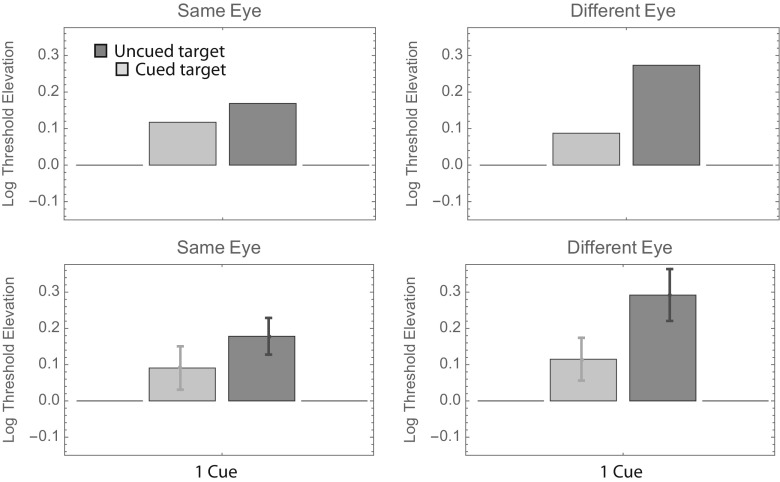
Results of Experiment 2, summarized in two ways. The format is analogous to that in Fig. 3.

As for Fig. 3, we pooled the data from Experiment 2 across observers in two ways. The top panels in Fig. 6 show weighted average log threshold elevations across observers, in which the weights are proportional to the inverse of the standard error. The bottom panels show the unweighted averages ± 1 standard error. There is quite a bit of similarity between these summaries and those appearing in Fig. 3. First, consider the different-eye (right-hand) panels. Recall that the timing in these conditions was identical to that in Experiment 1. Moreover, the monitor luminance and target frequency were identical to those used by observers M.L. and P.L.C. in Experiment 1. Thus, for these two observers, the 1-cue conditions in the two experiments were exactly the same. Consequently, it is reassuring to see that the average threshold elevations for these conditions (i.e., those shown in Figs. 3 and 6) are also within the measurement error of these experiments: The threshold elevation (i.e., above that for 100%-validly cued targets) was ~ 0.1 log units for (noninformatively) cued targets and ~ 0.2 log units for uncued targets. A more complete analysis of test–retest reliability using these observers’ data appears in the section below subtitled Further Analyses.

Also as in Experiment 1, Fig. 6 indicates an average threshold elevation of ~ 0.15 log units when uncued targets appear in the same eye as the cues. However, unlike Experiment 1, Fig. 6 indicates an average threshold elevation of ~ 0.1 log units when cued targets appear in the same eye as the cues. Indeed, a fully factorial, two-way ANOVA (Same or Different Eye × Cued or Uncued Target) with each observer’s threshold elevation (with respect to the 100%-valid same-eye condition) treated as a repeated measurement suggests only one significant effect: that of cued versus uncued target (*p* = .05). The main effect for same versus different eye was not significant (*p* > .25), nor was the interaction (*p* > .4). Since there were no 0-cue conditions in Experiment 2, we cannot use these data to confirm Experiment 1’s result that all targets (even the uncued ones) received some facilitation from our precues. Nor can we quantify how much less effective the precues were when they and the target were delivered to different eyes. However, we must note that the average difference between the same-eye and different-eye thresholds in Experiment 2 was smaller than Experiment 1’s difference of 0.115 log units. Here it was just 0.069 log units.

**Fig. 7:**
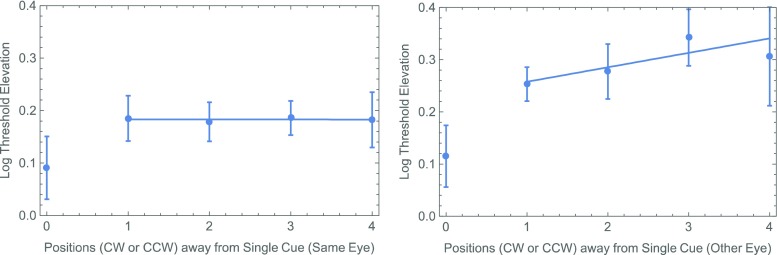
Summary of thresholds with a single noninformative cue from Experiment 2. The format is analogous to that in Fig. 4.

One of the reasons for running Experiment 2 was to see whether we could replicate Fig. 4’s surprising suggestion of a proximity effect between the target and a precue only when the latter appeared in the other eye. When the data were segregated on this basis (Fig. 7), there again does seem to be a systematic increase in threshold with the distance between a single cue and a different-eye target. However, once again, when the thresholds for cued targets (zero separation) are excluded, the effect is not significant: *F*(1, 26) = 1.19, *p* = .28. We saw no hint of any such effect with same-eye targets (Fig. 7, left panel): *F*(1, 26) = 0.00, *p* = .997.

We have no explanation for the effect of proximity in the different-eye data from Experiment 1, nor for the hint thereof from Experiment 2. The main difference between the results of Experiment 1 and those of Experiment 2 is the threshold elevations for noninformatively cued same-eye targets. Note that this difference is not huge: just 0.07 log units, on (unweighted) average, well within two standard errors of this measurement. Thus, one possible reason for this difference is sampling error. There are at least two other possible reasons, however: The CTOA was just 0.100 s in Experiment 2 (for same-eye targets), whereas it was 0.117 s in Experiment 1; also, only 16 conditions were interleaved in Experiment 2, whereas 32 were interleaved in Experiment 1.

Obvious choices for distinguishing these possibilities included a rerun of Experiment 1 with Experiment 2’s timing or a rerun of Experiment 2 with Experiment 1’s timing. The latter seemed the more prudent option, because the result consistent with the timing explanation above would be “positive” (i.e., smaller threshold elevations for noninformatively uncued same-eye targets) rather than “negative” (i.e., similar threshold elevations for cued and uncued targets). Furthermore, since there was no obvious reason for replicating the different-eye results with the same timing used in the first two experiments, we opted to extend its CTOA and see whether that would suffice to eradicate the threshold elevation for cued different-eye targets.

## Experiment 3

### Method

All four observers from Experiments 1 and 2 participated in Experiment 3. Again, only the two authors were aware of this study’s purpose. This experiment was identical to Experiment 2 in every way, except for the timing between the cue and target. See Fig. 1.

### Results

**Fig. 8:**
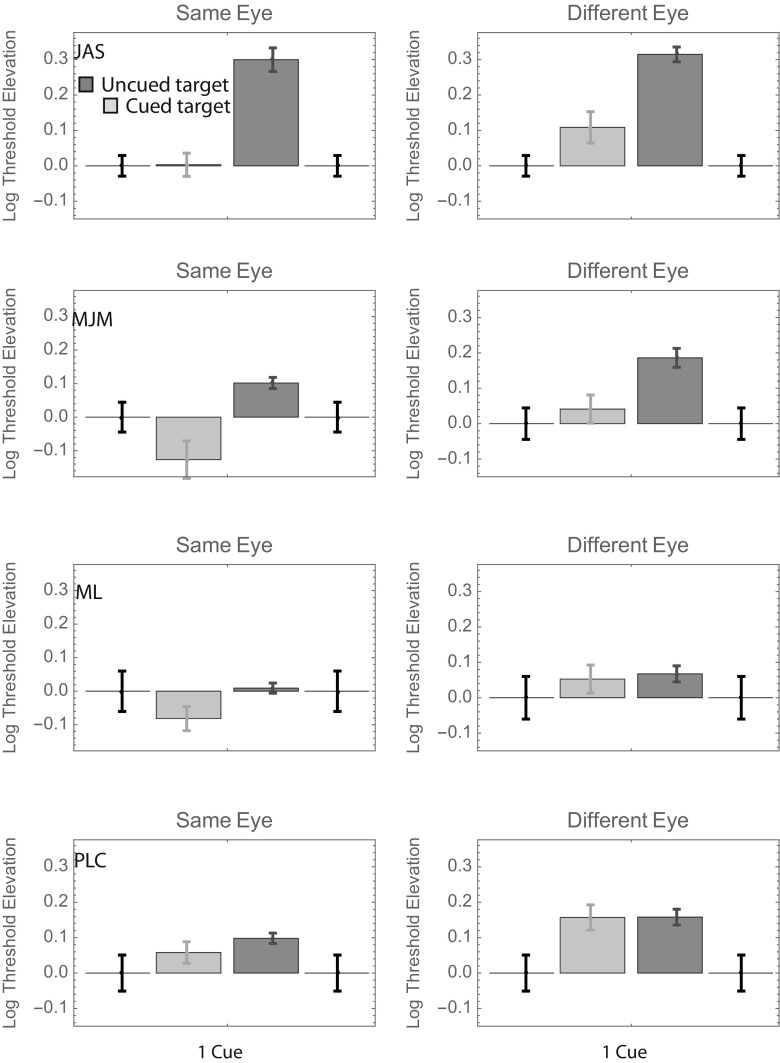
Log threshold contrast elevations with respect to the 100%-valid same-eye condition in Experiment 3. The format is analogous to that in Figs. 2 and 5: Different color bars show the results from conditions in which the target appeared at precued and uncued positions.

As for Figs. 2 and 5, we pooled the data from Conditions 2–8 when computing the uncued bar for each left panel of Fig. 8. Similarly, the data from Conditions 10–16 were pooled when computing the uncued bar for each right panel. As in Experiments 1 and 2, here it would be fair to say that most of the uncued bars are taller than their corresponding cued bars (although this difference is nonsignificant in two of the eight cases—a different two cases than in Exp. 2), and most different-eye bars are taller than most same-eye bars, although the difference is pretty small in some cases.

**Fig. 9:**
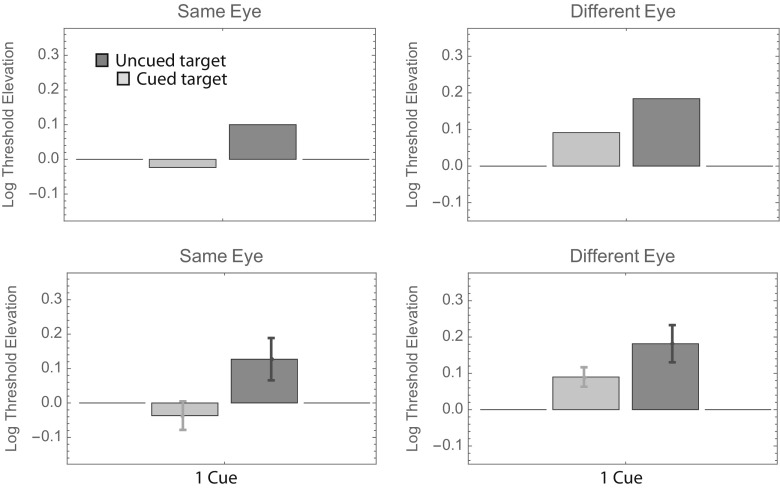
Results of Experiment 3, summarized in two ways. The format is analogous to that in Figs. 3 and 6.

As for Figs. 3 and 6, we pooled the data from Experiment 3 across observers in two ways. The top panels in Fig. 9 show weighted average log threshold elevations across observers, where the weights are proportional to the inverse of the standard error. As in Figs. 3 and 6, average thresholds are greatest in the uncued different-eye condition. Average thresholds are lowest in the cued same-eye condition, and indeed, these thresholds are no greater than those obtained in the 100%-valid (i.e., baseline) condition.

**Fig. 10:**
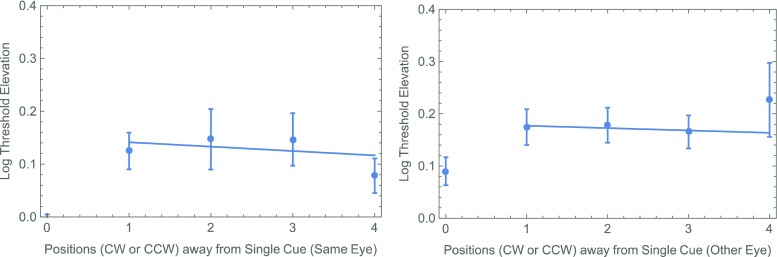
Summary of thresholds with a single noninformative cue from Experiment 3. The format is analogous to that in Figs. 4 and 7.

As in Experiment 2, a fully factorial, two-way ANOVA with each observer’s threshold elevation treated as a repeated measurement suggested a significant main effect of the cue (*p* < .02). The main effect of eye (an average difference of 0.090 log units) was not significant (*p* = .08), but it was close. The analysis produced no suggestion of an interaction (*p* > .4). Finally, the results from Experiment 3 also showed no suggestion of an effect between the position of the precue and that of an uncued target in either eye (see Fig. 10).

The relatively low thresholds for cued same-eye targets in Experiment 3 are at least broadly consistent with the theory that 0.100 s between the cue and target onsets might not have been quite enough for the full benefit of a noninformative precue (cf. the CTOAs of 0.117 s in Exp. 1 and 0.108 s in Solomon, 2004). On the other hand, the use of group-level statistics may not necessarily be appropriate when there are qualitative individual differences. For subjects J.A.S. and P.L.C., the contrast thresholds for intra-ocularly cued targets were similarly low, regardless of whether those cues were 100% valid or noninformative. The same result was obtained for subject M.L., but only with a CTOA of 0.117 s. When the CTOA was 0.100 s, his results were qualitatively more similar to those of M.J.M., who derived an additional benefit (of ~ 0.1 log unit) when targets were intra-ocularly cued with 100% validity. Thus, we concluded that the main difference between the results of Experiments 1 and 2 can be attributed to a single observer (M.L.) who derived less benefit from the noninformative intra-ocular cue in Experiment 2.

## Experiments 1–3: Further analyses

**Fig. 11:**
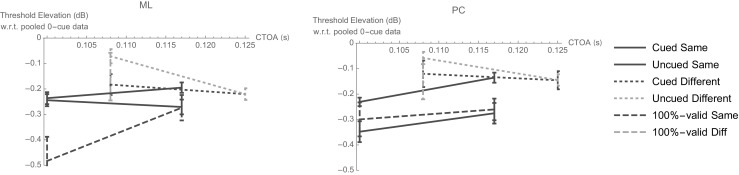
Log threshold contrast elevations with respect to the 0-cue condition in Experiment 1, plotted against cue–target onset asynchrony (CTOA). Error bars show the square root of the sum of the squared standard errors from each experiment.

Experiments 2 and 3 were conducted to ensure that the greater elevation of thresholds with interocular cues in Experiment 1 was not simply an artifact of their slightly shorter CTOAs. Consequently, it seemed logical to plot comparative results from all three experiments as a function of CTOA. Remember that only two of our observers performed all three experiments with the same stimuli. Their results are strikingly similar when expressed as threshold elevations with respect to the 0-cue condition in Experiment 1 (see Fig. 11).

Both of these subjects experienced benefits between 0.15 and 0.20 log units (over the 0-cue condition) from different-eye cues with a CTOA of 0.125 s, regardless of whether or not the target was cued. Similar benefits were also obtained from different-eye cues with a CTOA of 0.108 s, regardless whether that cue was 100% valid or noninformative. The benefits were smaller (~ 0.05 log units) for interocularly uncued targets with this CTOA. Benefits between 0.15 and 0.20 log units were also obtained from different-eye cues with a CTOA of 0.117 s. The benefits were even greater (~ 0.25 log units) for intra-ocularly uncued targets with a CTOA of 0.100 s. Both subjects also enjoyed a benefit of ~ 0.3 log units for intra-ocularly cued targets with a CTOA of 0.117 s, regardless of whether that cue was 100% valid or noninformative. The 100%-valid cues were even more beneficial with a CTOA of 0.100 s, although the extra benefit was significant only for M.L. The only notable difference between the observers was with respect to the benefit of noninformative cues with a CTOA of 0.100 s. On the basis of these data alone, it is impossible to draw any firm conclusions: P.C.’s benefit was similar to that obtained with 100%-valid cues, but M.L.’s was not. J.A.S.’s data were qualitatively similar to P.C.’s in this regard (results not shown, but his cued same-eye thresholds in Exps. 2 and 3 were all within 0.005 log units of each other, regardless of whether the cue was 100% valid or noninformative). However, M.J.M.’s data were qualitatively similar to M.L.’s, at least in Experiments 2 and 3: His same-eye thresholds for noninformatively cued targets were ~ 0.1 log units higher than his same-eye thresholds in both experiments’ 100%-valid conditions.

Individual differences aside, it seems fair to say that all observers enjoyed a benefit from both types of precue (100% valid and noninformative), even with interocular (i.e., different-eye) cues, and even when the target itself was not cued. There was no general trend toward or away from larger cueing effects as the CTOA increased from 0.100 to 0.125 s. Also, at the group level, the thresholds for similar combinations of cue, eye, and CTOA were no different in Experiments 2 and 3 than those obtained in Experiment 1 [data not shown; paired *t* test with *t*(9) = 1.6, *p* = .13].

## General discussion

The same-eye results of Experiment 1 confirm previous claims (Solomon, 2004; Wright, 1994) that multiple precues can facilitate processing at multiple positions just as well as a single precue. Moreover, we now know that maximum facilitation requires the cue and target to be delivered to the same eye.

Self and Roelfsema (2010) also reported that multiple (in their case, two) interocularly presented precues could facilitate orientation identification just as well as one (average response times for the “cued, across” and “cued, within” conditions were similar in their Exp. 2, and were identical with 0.150-s CTOAs). However, they argued *against* unlimited capacity for two reasons, both of which we find unconvincing. Their first reason is that the response times for monocularly presented targets were somewhat longer following binocularly presented precues than following monocular cues presented to the target’s eye. This result, of course, does indicate some kind of capacity limit, but not necessarily at the monocular level. Indeed, we believe it would be consistent with the “monocular competition” advocated by Self and Roelfsema elsewhere in their article. Simply put, the average response times obtained with binocular precues quite plausibly reflect the average of the response times obtained with monocular precues presented to the two eyes (i.e., the target’s eye and the other eye).

Self and Roelfsema’s (2010) second argument against unlimited capacity for facilitatory precueing stems from the implicit assumption that such precueing must be mediated by local sensory interactions. Thus, their failure to find any effect of cue size (and thus of its distance from the target) on response times (specifically those for targets presented to the same eye as the precue) was used not only to justify attributing their shorter response times to something other than local sensory interactions, but also to justify labeling the monocular-level mechanism “attentional” rather than “preattentive.”

Like Self and Roelfsema (2010), we found no effect of the distance between a monocularly delivered precue and an uncued target delivered to the same eye. However, there do seem to be effects of proximity on performances with uncued targets following both binocular cues (Self & Roelfsema, 2010) and different-eye cues (our Exp. 1). Consequently, we agree with their conclusion in favor of a retino-topic “zoom-lens” associated with higher-level attentional selection. The absence of any proximity effect with the relatively long CTOAs in our Experiment 3 may be indicative of the speed with which attention can focus on a specific position; however, the experiment was not designed specifically to investigate that question. Other experiments with binocularly delivered cues and targets (e.g., Popple & Levi, 2005; Self & Roelfsema, 2010; Solomon & Morgan, 2001) suggest a somewhat longer time scale.

Despite the similarity of our findings to those of Self and Roelfsema (2010), there are some notable differences. Whereas we found only main effects of cue (target cued vs. target uncued) and eye (same vs. different eye), they also reported a significant interaction between these independent variables. At this point, we cannot be certain whether this discrepancy can be attributed to the difference between our dependent variables (we measured threshold contrast; they measured response times) or their inclusion of very brief (0.050-s) CTOAs. (Inspection of their Fig. 2 suggests that their inclusion of longer CTOAs cannot be responsible for the interaction.)

It should be noted that although our results convincingly implicate an unlimited capacity for the effect of multiple *precues* on visual sensitivity, they say nothing about observers’ capacity for making decisions about (or between) multiple *targets*. The latter ability does seem to be quite limited (see, e.g., Sperling, 1960).

We speculate that exogenous cueing (a *procedure*) can lead to a fast and automatic *process*, which is perhaps ordinarily but not necessarily used in conjunction with the (capacity-limited) attentional selection of information from specific positions that has been studied in experiments on full and partial report (e.g., Sperling, 1960), serial search (e.g., Neisser, 1967), and change blindness (e.g., Rensink, O’Regan, & Clark, 1997).

Solomon (2004) suggested that this process might effectively increase the gain of mechanisms sensitive to the difference between potential target tilts, and that this increase in gain is transient; it need not also extend to each trial’s epoch of poststimulus masking (cf. Carrasco & McElree, 2001). Our present results are consistent with this general idea. We suggest that a better term for the process would be the physiological term “sensitization” (e.g., Squire & Kandel, 1999), because the latter carries no implication that attention is involved or that information is being transferred.

## Electronic supplementary material

ESM 1 (DOCX 631 kb)

## Footnotes

1 A detailed document describing these estimates has be made available as supplementary material.
