# Supplementary material for: Precues’ elevation of sensitivity is not only preattentive, but largely monocular
Source: Atten Percept Psychophys. 2018 Jul 9;80(7):1705–17. doi: 10.3758/s13414-018-1564-1 (PMC6153965; doi:10.3758/s13414-018-1564-1)
Supplement: Supplementary file 1 — (DOCX 631 kb) [file 13414_2018_1564_MOESM1_ESM.docx]

**Supplement: Estimates of psychometric slopes in all experiments**

***Experiment 1***

Maximum-likelihood estimates of psychometric slope for each observer are shown, with their corresponding standard errors, in Fig. S1. (Columns grow from the value for slope in the 100%-valid same-eye condition.) Red and blue bars show slopes obtained when the target did and did not appear at a cued position.

Visual inspection of Fig. S1 reveals that most bars tend to decrease from the (100%-valid same-eye) baseline. Beyond that, it is hard to discern any overall pattern that is common to all observers. To facilitate such an analysis, we have pooled the data across observer in two ways. The top panels in Fig. S2 show weighted average log slope across observers, where the weights are proportional to the inverse of the standard error. The bottom panels show the unweighted averages ± 1 standard error. Both summaries suggest shallower slopes with noninformativeprecues, compared to the 100%-valid, same-eye baseline. However, neither of Fig. S2’s summaries (nor those in Fig. 3 of the main paper) suggest any appreciable or significant difference between this baseline condition’s slope (and threshold) and the slope (and threshold) obtained with one noninformative, same-eye precue.

Figure S2’s summaries suggest that uncued targets produced shallower psychometric functions than cued targets. Whereas this main effect of cue validity achieved statistical significance (*p* = .04) in a fully factorial, three-way ANOVA (Same or Different Eye × Number of Pre-Cues × Cued or Uncued Target) with the logarithm of each observer’s maximum-likelihood estimates of slope treated as a repeated measurement, none of the other main effects or interactions did (all *p*s > .11).

Any effect of cue–target distance on uncued thresholds, such as that suggested by Fig. 4 in the main paper, must necessarily result in a reduction of psychometric slope when data are pooled across cue–target distances. There is no reason to suspect any relationship between cue–target distance and psychometric slope itself, but we have performed the analysis anyway (see Fig. S3). M.J.M.’s data were excluded from this analysis because, in several cases, they did not adequately constrain estimates of slope. The take-home message from Fig. S3 is that there is no consistent difference between slopes with cued targets and those with uncued targets, when data are segregated on the basis of cue–target distance.

***Experiment 2***

As we did when creating Fig. S1, we pooled the data from Conditions 2–8 when computing the blue bar for each left panel of Fig. S4. Unlike most of the bars in Fig. S1, only about half of the bars in Fig. S4 decrease from the (100%-valid same-eye) baseline.

As we did when creating Fig. S2, we pooled the data from Experiment 2 across observer in two ways. The top panels in Fig. S5 show weighted average log slope across observers, where the weights are proportional to the inverse of the standard error. The bottom panels show the unweighted averages ± 1 standard error.

Like Fig. S2, Fig. S5’s summaries suggest that uncued targets produced shallower psychometric functions than cued targets. As in Experiment 1, here this main effect of cue validity achieved statistical significance (*p* = .01) in a fully factorial, two-way ANOVA (Same or Different Eye × Cued or Uncued Target) with the logarithm of each observer’s maximum-likelihood estimates of slope treated as a repeated measurement. Neither the other main effect nor the interaction did (both *p*s > .46).

As we did when creating Fig. S3, we segregated data on the basis of cue–target distance for Fig. S6. M.J.M.’s data were excluded once again, because they did not adequately constrain some estimates of slope. Neither of the regression lines in Fig. S6 illustrates a significant effect of cue–target distance on psychometric slope for uncued targets (both *p*s > .3). However, those slopes are uniformly lower than the corresponding slopes for cued targets (cf. the take-home message from Fig. S3).

***Experiment 3***

As we did when creating Figs. S1 and S4, we pooled the data from Conditions 2–8 when computing the blue bar for each left panel of Fig. S7. As in Fig. S4, approximately half of the bars in Fig. S7 decrease from the (100%-valid same-eye) baseline.

As we did when creating Figs. S2 and S5, we pooled the data from Experiment 3 across observer in two ways. The top panels in Fig. S8 show weighted average log slope across observers, where the weights are proportional to the inverse of the standard error. The bottom panels show the unweighted averages ± 1 standard error.

Like Figs. S2 and S5, Fig. S8’s summaries suggest that uncued targets produced shallower psychometric functions than cued targets. As in Experiments 1 and 2, here this main effect of cue validity achieved statistical significance (*p* = .007) in a fully factorial, two-way ANOVA (Same or Different Eye × Cued or Uncued Target) with the logarithm of each observer’s maximum-likelihood estimates of slope treated as a repeated measurement. Neither the other main effect nor the interaction did (both *p*s > .38).

As we did when creating Figs. S3 and S6, we segregated data on the basis of cue–target distance for Fig. S9. M.J.M.’s data were included this time. Neither of the regression lines in Fig. S9 illustrates a significant effect of cue–target distance on psychometric slope for uncued targets (both *p*s > .48). In this case, uncued slopes are lower than cued slopes only for the different-eye conditions.

**Fig. S1** Psychometric slopes in Experiment 1. Red and blue bars show results from conditions in which the target appeared at precued and uncued positions, respectively. Error bars contain maximum-likelihood estimates ± 1 *SD* from the parametric bootstrap distribution. Horizontal lines and gray error bars are associated with the 100% valid, same-eye condition. (Results from the 100%-valid different-eye condition are not shown.)

**Fig. S2** Psychometric slopes from Experiment 1, summarized in two ways. In the top two panels, each bar shows the weighted average log threshold elevation across observers. Weights are proportional to the inverse of the standard error. In the bottom two panels, each bar shows the unweighted average log threshold elevation across observers. Error bars show ± 1 *SE* (i.e., ± 1 *SD*/$\sqrt{N}$, where *N* = 4).

**Fig. S3** Summary of slopes with a single non-informative cue from Experiment 1. Each of these points represents the unweighted average (± 1 standard error) across observers J.A.S., M.L., and P.C. Straight lines are (least-squares) best-fits through the data from uncued positions.

**Fig. S4** Psychometric slopes in Experiment 2. The format is analogous to that in Fig. S1: Red and blue bars show results from conditions in which the target appeared at precued and uncued positions, respectively.

**Fig. S5** Psychometric slopes from Experiment 2, summarized in two ways. The format is analogous to that in Fig. S2.

**Fig. S6** Summary of slopes in Experiment 2. Each of these points represents the unweighted average (± 1 standard error) across observers J.A.S., M.L., and P.C. Straight lines are (least-squares) best-fits through the data from uncued positions.

**Fig. S7** Psychometric slopes in Experiment 3. The format is analogous to that in Figs. S1 and S4: Red and blue bars show results from conditions in which the target appeared at precued and uncued positions, respectively.

**Fig. S8** Psychometric slopes from Experiment 3, summarized in two ways. The format is analogous to that in Figs. S2 and S5.

**Fig. S9** Summary of slopes in Experiment 3. Each of these points represents the unweighted average (± 1 standard error) across observers J.A.S., M.L., P.C., and M.J.M. Straight lines are (least-squares) best-fits through the data from uncued positions.
